# Prognostic value of lymphocyte count for in-hospital mortality in patients with severe AECOPD

**DOI:** 10.1186/s12890-022-02137-1

**Published:** 2022-10-05

**Authors:** Yanlu Hu, Huanyu Long, Yang Cao, Yanfei Guo

**Affiliations:** 1 Department of Respiratory and Critical Care Medicine, Beijing Hospital, National Center of Gerontology; Institute of Geriatric Medicine, Chinese Academy of Medical Sciences, Beijing, P.R. China; 2 The First Affiliated Hospital of Jinan University (Also Known as Guangzhou Overseas Chinese Hospital and the First Clinical Medical College of Jinan University), Guangzhou, People’s Republic of China

**Keywords:** Exacerbation, Chronic obstructive pulmonary disease, Lymphocyte count, Mortality, Biomarker

## Abstract

**Background:**

Patients with severe acute exacerbations of chronic obstructive pulmonary disease often have a poor prognosis. Biomarkers can help clinicians personalize the assessment of different patients and mitigate mortality. The present study sought to determine if the lymphocyte count could act as a risk factor for mortality in individuals with severe AECOPD.

**Methods:**

A retrospective study was carried out with 458 cases who had severe AECOPD. For analysis, patients were divided into two groups on the basis of lymphocyte count: < 0.8 × 10^9^/L and ≥ 0.8 × 10^9^/L.

**Results:**

Patients who fulfilled the criteria for inclusion were enrolled, namely 458 with a mean age of 78.2 ± 8.2 years. Of these patients, 175 had a low lymphocyte count. Compared to patients with normal lymphocyte counts, those with low counts were older (79.2 ± 7.4 vs. 77.5 ± 8.6 years, *p* = 0.036), had lower activities of daily living scores on admission (35.9 ± 27.6 vs. 47.5 ± 17.1, *p* < 0.001), and had a greater need for home oxygen therapy (84.6 vs. 72.1%, *p* = 0.002). Patients with low lymphocytes had higher mortality rates during hospitalization (17.1 vs. 7.1%, *p* = 0.001), longer hospital stay (median [IQR] 16 days [12–26] vs. 14 days [10–20], *p* = 0.002) and longer time on mechanical ventilation (median [IQR] 11.6 days [5.8–18.7] vs. 10.9 days [3.8–11.6], *p* < 0.001). The logistic regression analysis showed lymphocyte count < 0.8 × 10^9^/L was an independent risk factor associated with in-hospital mortality (OR 2.74, 95%CI 1.33–5.66, *p* = 0.006).

**Conclusion:**

Lymphocyte count could act as a predictor of mortality in patients with severe AECOPD.

**Supplementary Information:**

The online version contains supplementary material available at 10.1186/s12890-022-02137-1.

## Background

Chronic obstructive pulmonary disease (COPD) is a global epidemic with a high incidence of morbidity and mortality, often resulting in a poor prognosis for patients. Acute exacerbation of COPD (AECOPD) occurs when respiratory problems get worse, resulting in the need for further clinical treatment, and it often becomes a critical condition with poor prognosis [1]. At present, according to the World Health Organization (WHO), COPD is the third leading cause of death in the world [2]. The latest epidemiological survey of COPD in China shows that the prevalence of COPD among people aged 40 and above is as high as 13.7%, and there are currently an estimated 100 million cases of COPD in China [3]. AECOPD is one of the leading causes of hospitalization, which significantly increases the mortality rate of AECOPD patients [4]. Early identification of risk factors for poor prognosis can help stratify patient management and reduce mortality, readmission rates and the socioeconomic burden [5].

Patients with AECOPD have significant individual differences, and it is sometimes difficult for clinicians to perform an accurate prognostic analysis. Early identification of risk factors associated with poor prognosis in AECOPD can effectively help clinicians to develop individualized treatment plans for patients with AECOPD. Old age, dyspnea and having comorbidities have been shown to be predictors of poor prognosis for AECOPD [6–8]. There is still a need for more biomarkers in clinical practice to analyze the condition of AECOPD patients and intervene early in the prognosis.

Lymphocytes are associated with human immune function and inflammatory status [9]. Currently, the predictive role of lymphocytes on the prognosis of AECOPD is unclear and fewer studies have been conducted in this field. In a three-year prospective study, Acanfora found that a relative lymphocyte count ≤ 20% was an independent risk factor for mortality within three years in elderly patients with severe COPD [10]. However, lymphocyte percentage was influenced by other leukocyte subpopulations, and other outcome variables in patients with AECOPD were not described in the study.

Accordingly, we aimed to elucidate the association between low lymphocyte count and in-hospital mortality, length of stays and use time on ventilator during hospitalization in severe AECOPD patients.

## Methods

### Study design and patients

Patients aged 40 years and above who were admitted to Beijing Hospital for treatment of severe AECOPD from January 2011 to September 2021 were included. AECOPD was considered aggravated dyspnea with an increase in cough and/or amount of sputum or its purulent appearance, needing more care [1]. All diagnoses, namely the primary and five secondary diagnoses, were based on the International Classification of Diseases, 10th Revision (ICD10) coding system. Exclusion criteria for the study were length of stay of less than 24 h or readmission within one month. And cases who had been admitted for AECOPD in the month prior to the current admission were also excluded. Our study was conducted in accordance with the Declaration of Helsinki and with approval from the Ethics Committee of Beijing Hospital (BJ-2018-199).

In this study, severe AECOPD was defined as AECOPD requiring admission to the intensive care unit (ICU) and to the general respiratory ward during hospitalization with a diagnosis of respiratory failure or requiring mechanical ventilation. The patient's laboratory test results were obtained from the first examination within 24 h of admission.

In this study, lymphopenia was defined as the absolute count below 0.8 × 10^9^/L. We chose this cut-off value because most Chinese laboratories use 0.8 × 10^9^/L as the lower limit of normal lymphocyte values [11].

### Data collection

The baseline data obtained from all enrolled patients were extensive, including demographic characteristics, complete blood count, metabolic indices, arterial blood gases and comorbidities. Demographics included age, sex, body mass index, smoking status, length of stay, use of long-term home oxygen therapy, index of activities of daily living (ADL) at admission, exacerbation history, medication before admission, heart rate, body temperature, blood pressure, requirement for invasive mechanical ventilation (IMV) and time on ventilator. Medication before admission means taking antibiotics and steroids as advised by the community clinic within 72 h prior to this hospitalization. Blood tests included red cell count (RBC), total and differential white blood cell counts (WBC), neutrophil-to-lymphocyte ratio (NLR), platelet-to-lymphocyte ratio (PLR), C reactive protein (CRP), N-terminal pro-brain natriuretic peptide (NT-proBNP), D-dimers, creatinine and uric acid. Blood tests was obtained in the first 24 h after admission. The following comorbidities were included in this study: respiratory failure, coronary heart disease (CHD), chronic heart failure (CHF), hypertension, atrial fibrillation, chronic kidney disease (CKD), diabetes, gastroesophageal reflux (GER), anemia and obstructive sleep apnea hypopnea syndrome (SAHS).

### Statistical analysis

The statistical software used for the study was SPSS 26.0. Results were presented as mean ± standard deviation (SD) or median (interquartile range [IQR]) as appropriate. And categorical variables were characterized using percentages. Continuous variables were tested with t-tests and categorical variables with chi-square tests to determine significant differences between groups. The Mann-Witney U test was used for comparison of length of stays and duration of mechanical ventilation.

We used univariate and multivariate analyses in logistic regression to determine whether lymphopenia was associated with in-hospital mortality. Variables with *p*-values < 0.2 in univariate analysis were eventually included in multivariate logistic regression analysis. *p*-values < 0.05 were considered to be statistically significant.

## Results

The initial number of study patients was 464, but 6 were excluded because of lack of information on lymphocyte count. The 458 individuals making up the final sample had a mean age of 78.2 ± 8.2 years and consisted of mostly males (72.9%). There were 50 patients (10.9%) who died during hospitalization, and 408 patients (89.1%) were discharged. The demographic characteristics of survivors and non-survivors, as well as laboratory results, are shown in Additional file 1: Table S1. Patients with in-hospital death had lower ADL scores on admission, a higher rate of combined heart failure and the requirement for IMV during hospitalization. And patients in the in-hospital death group also had higher white blood cell counts and lower lymphocyte counts. Compared with the survivor group, more patients in the death group had lymphocyte counts < 0.8 × 10^9^/L. (60% vs. 35.5%, *p* = 0.001).

In the overall population, 175 patients (38.2%) had lymphocyte counts < 0.8 × 10^9^/L and 283 patients (81.8%) had normal lymphocyte counts. The demographic and clinical characteristics of the patients in each group are shown in Table 1. The age of patients with low lymphocytes was (79.2 ± 7.4) years, which was significantly higher than the other group (77.5 ± 8.6 years, *p* = 0.036). About 28.0% of patients with low lymphocytes were on long-term home oxygen therapy, a higher percentage than in the normal lymphocyte group (19.1%, *p* = 0.026). And patients with low lymphocytes had lower ADL scores on admission (35.9 ± 27.6 vs. 47.5 + 17.1, *p* < 0.001). Heart rate was higher in the lymphopenia group and more patients in the low lymphocyte group had combined respiratory failure, a higher percentage than in the other group (84.6% vs. 72.1%, *p* = 0.002). Patients with low lymphocyte counts had higher rates of combined diabetes, CKD, and anemia compared to patients in the normal lymphocyte group.

**Table 1:** Clinical features and comorbidities in all patients included in two groups

Variables	Total (N = 458)	Lymphocyte count < 0.8 × 10^9^/L (N = 175)	Lymphocyte count > 0.8 × 10^9^/L (N = 283)	*p* value
Age, years	78.2 (8.2)	79.2 (7.4)	77.5 (8.6)	0.036
Male	334 (72.9%)	131 (74.9%)	203 (71.7%)	0.464
Smoking status				0.115
Never smoker	110 (20.0%)	38 (21.7%)	72 (25.4%)	
Former smoker	292 (63.8%)	131 (74.9%)	161 (56.9%)	
Current smoker	56 (12.2%)	20 (11.4%)	36 (12.7%)	
BMI, kg/m^2^	23.2 (4.7)	22.7 (4.7)	23.5 (4.7)	0.060
Long-term home oxygen therapy	103 (22.5%)	49 (28.0%)	54 (19.1%)	0.026
ADL index at admission	43 (29.7)	35.9 (27.6)	47.5 (30.1)	< 0.001
Exacerbation history in the past year	197 (43.0%)	76 (43.4%)	121 (42.8%)	0.888
Regular use of long-acting bronchodilators	105 (22.9%)	47 (26.8%)	58 (20.5%)	0.116
Oral steroid before admission	45 (9.8%)	19 (10.9%)	26 (9.2%)	0.560
Oral antibiotics before admission	99 (21.6%)	44 (25.1%)	55 (19.4%)	0.149
Requirement for IMV	64 (14.0%)	31 (17.7%)	33 (11.7%)	0.069
Body temperature, °C	36.5 (0.5)	36.6 (0.6)	36.5 (0.5)	0.239
Pulse, bpm	89.4 (15.8)	91.7 (16.4)	87.9 (15.2)	0.012
Respiratory rate, bpm	21.4 (5.4)	22.0 (5.0)	21.0 (5.6)	0.056
Systolic pressure, mmHg	134.1 (20.2)	134.8 (21.7)	133.7 (19.3)	0.555
Diastolic pressure, mmHg	72.9 (11.5)	72.6 (11.7)	73.0 (11.4)	0.750
Comorbidities				
Respiratory failure	352 (76.9%)	148 (84.6%)	204 (72.1%)	0.002
Hypertension	265 (57.9%)	96 (54.z9%)	169 (59.7%)	0.306
CHD	141 (30.8%)	49 (28.0%)	92 (32.5%)	0.310
CHF	119 (26.0%)	54 (30.9%)	65 (23.0%)	0.061
Atrial fibrillation	86 (18.8%)	38 (21.7%)	48 (17.0%)	0.206
Diabetes	115 (25.1%)	53 (30.8%)	62 (21.9%)	0.045
CKD	71 (15.5%)	36 (20.6%)	35 (12.4%)	0.018
GER	82 (17.9%)	36 (20.6%)	46 (16.3%)	0.242
Anemia	55 (12.0%)	30 (17.1%)	25 (8.8%)	0.008
SAHS	30 (6.6%)	8 (4.6%)	22 (7.8%)	0.178

Date is presented as mean ± standard deviation (SD) for continuous variables and percentages for categorical variables

*BMI* body mass index, *IMV* invasive mechanical ventilation, *CHD* coronary heart disease, *CHF* chronic heart failure, *CKD* chronic kidney diseases, *GER* gastroesophageal reflux, *SAHS* sleep apnea syndrome

Table 2 shows the results of the first laboratory tests performed within 24 h of admission. Patients with lymphocyte count of < 0.8 × 10^9^/L had a lower platelet count (171.2 ± 69.8 vs. 204.4 ± 113.5 × 10^9^/L, *p* = 0.001) and lower eosinophil count (61.3 ± 132.5 vs. 145.4 ± 198.4 × 10^6^ /µL, *p* < 0.001) compared to patients with normal lymphocyte count. Patients with low lymphocytes had a significantly higher NLR (18.6 ± 22.8 vs. 5.6 ± 6.2, *p* < 0.001) and PLR (421.4 ± 497.8 vs. 164.5 ± 106.2, *p* < 0.001) than did the normal lymphocyte group. And patients in the low lymphocyte count group had a higher CRP (7.3 ± 12.5 vs. 3.9 ± 4.8 mg/L, *p* = 0.001), blood glucose (8.0 ± 3.0 vs. 6.2 ± 2.1 mmol/L, *p* < 0.001) and creatine (93.4 ± 94.1 vs. 79.2 ± 36.6 µmol/L, *p* = 0.035) than the other group. Among the blood gas analysis parameters, the low lymphocyte group had lower pH (7.36 ± 0.08 vs. 7.38 ± 0.06, *p* = 0.001), higher PaCO_2_ (55.2 ± 17.4 vs. 51.1 ± 15.4 mmHg, *p* = 0.011) and lower oxygenation index (232.8 ± 107.9 vs. 254.5 ± 111.2 mmHg, *p* = 0.041).

**Table 2:** Laboratory results of patients within 24 h after admission

Variables	Total (N = 458)	Lymphocyte count < 0.8 × 10^9^/L (N = 175)	Lymphocyte count > 0.8 × 10^9^ /L (N = 283)	*p* value
White blood cell count, × 10^9^/L	8.3 (3.7)	8.3 (4.6)	8.3 (3.1)	0.939
Red blood cell count, × 10^9^/L	4.0 (0.7)	4.0 (0.7)	4.1 (0.7)	0.074
Platelet count, × 10^9^/L	191.7 (100.3)	171.2 (69.8)	204.4 (113.5)	0.001
Eosinophil count, × 10^6^ /uL	113.2 (180.7)	61.3 (132.5)	145.4 (198.4)	< 0.001
Neutrophil count, × 10^9^ /L	7.2 (7.3)	7.3 (4.6)	7.1 (8.5)	0.841
NLR, %	10.6 (16.2)	18.6 (22.8)	5.6 (6.2)	< 0.001
PLR, %	262.7 (341.8)	421.4 (497.8)	164.5 (105.2)	< 0.001
CRP, mg /L	5.2 (8.7)	7.3 (12.5)	3.9 (4.8)	0.001
D-dimers, ug/L	733.8 (1183.7)	822.1 (1184.5)	679.2 (1182.0)	0.210
NT-proBNP, pg/ml	1007.7 (2103.5)	1174.0 (2162.5)	904.9 (2063.3)	0.184
Blood glucose, mmol/L	6.9 (3.0)	8.0 (3.9)	6.2 (2.1)	< 0.001
Albumin, g/L	35.0 (5.3)	34.5 (6.0)	35.4 (4.8)	0.072
Fibrinogen, g/L	4.2 (3.3)	4.1 (1.5)	4.2 (4.0)	0.757
Creatinine, umol /L	84.7 (59.7)	93.4 (84.1)	79.2 (36.6)	0.035
Uric acid, umol /L	268.1 (110.3)	282.4 (141.0)	259.2 (111.2)	0.071
pH	7.37 (0.07)	7.36 (0.08)	7.38 (0.06)	0.001
PaO_2_, mmHg	78.8 (26.6)	80.5 (30.7)	77.8 (23.7)	0.288
PaCO_2_, mmHg	52.6 (16.3)	55.2 (17.4)	51.1 (15.4)	0.011
PaO_2_/FiO_2_, mmHg	246.23)	232.8 (107.9)	254.5 (111.2)	0.041
BE	3.6 (5.0)	3.8 (5.5)	3.4 (4.7)	0.483
HCO_3_^−^	29.1 (6.1)	29.2 (5.8)	29.1 (6.3)	0.852

Date is presented as mean ± standard deviation for continuous variable and percentages for categorical variables

*NLR* neutrophil/lymphocyte ratio, PLR platelet/lymphocyte ratio, CRP C reactive protein, *NT-proBNP* N-terminal pro-brain natriuretic peptide, *PaO*_*2*_ arterial partial pressure of oxygen, *PaCO*_*2*_ arterial partial pressure of carbon dioxide

Multivariate regression model showed lymphocytes < 0.8 × 10^9^/L were independent risk factors associated with in-hospital mortality (OR 2.74, 95%CI 1.33–5.66, *p* = 0.006; Table 3, Additional file 1: Table S2).

**Table 3:** Regression model of lymphopenia on in-hospital mortality in severe AECOPD

	Unadjusted OR (95%CI)	*p*	Adjusted OR (95%CI)	*p*
Lymphocyte count, × 10^9^/L				
< 0.8	2.72 (1.49–4.96)	0.001	2.74 (1.33–5.66)*	0.006
≥ 0.8	1.00 (ref)		1.00 (ref)	

*Adjusted for age, CHF, anemia, WBC, NLR, CRP, albumin, NT-proBNP, D-dimer, PaCO_2_, uric acid, requirement for IMV, the admission index of ADL

**Fig. 1:**
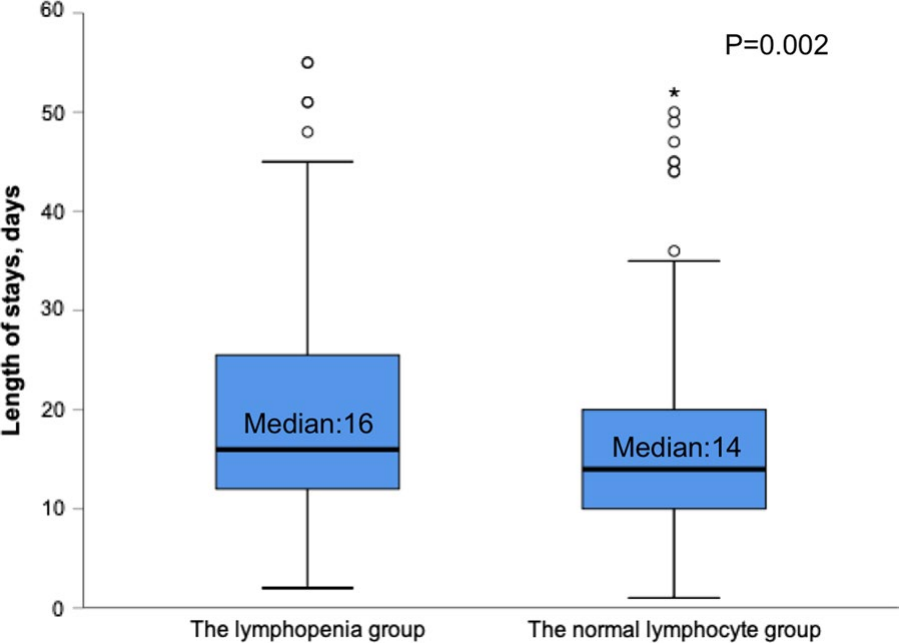
Length of stays for patients with severe AECOPD. AECOPD, acute exacerbation of chronic obstructive pulmonary disease. The circles and asterisks are outliers

**Fig. 2:**
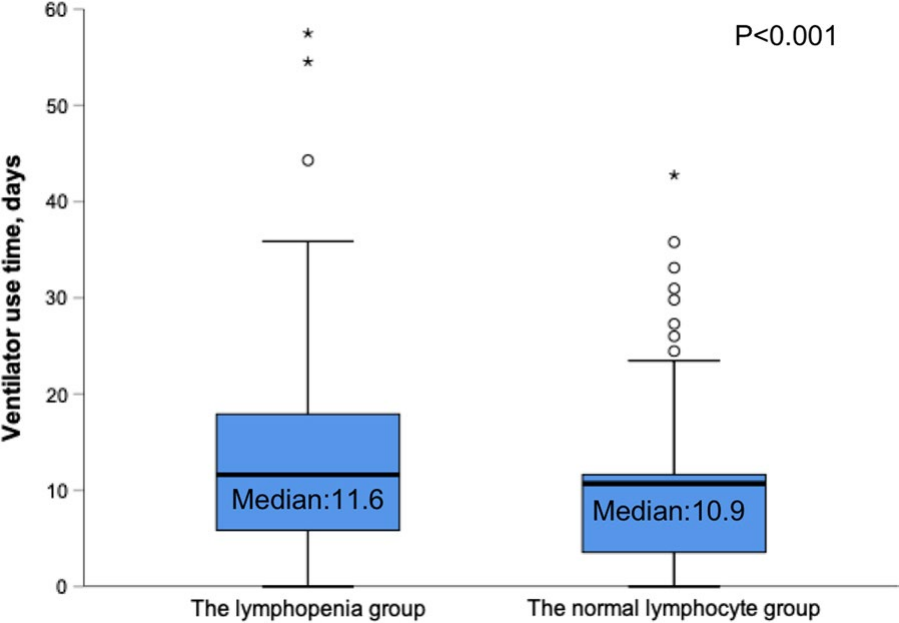
Ventilator use time for patients with severe AECOPD. AECOPD, acute exacerbation of chronic obstructive pulmonary disease. The circles and asterisks are outliers

In addition, the lymphopenia group had longer hospital stay (median [IQR] 16 days [12–26] vs. 14 days [10–20], *p* = 0.002; Fig. 1) and longer time on mechanical ventilation (median [IQR] 11.6 days [5.8–18.7] vs. 10.9 days [3.8–11.6], *p* < 0.001; Fig. 2).

## Discussion

We investigated the predictive value of low lymphocyte counts in patients with severe AECOPD. We found that patients in the low lymphocyte group were older, had more comorbidities, longer hospital stays, longer ventilation times, and higher in-hospital mortality. Lymphocyte count < 0.8 × 10^9^/L was an independent risk factor for in-hospital death in patients with severe AECOPD.

In recent years, there has been an increasing number of studies on biomarkers of prognosis in patients with AECOPD. Among them, the predictive value of NLR for the prognosis of patients with AECOPD has been studied more frequently. Some investigators have found that NLR has an independent influence on mortality during hospitalization and 28-day mortality in AECOPD patients [12–14]. NLR, however, is affected by both cell counts and does not show the predictive value as visually and directly as lymphocyte counts. In our previous paper, we found that lymphocyte count < 0.8 × 10^9^/L was an independent risk indicator for in-hospital death in AECOPD patients requiring ICU admission [15]. Xiong found that lymphocyte counts were lower in the stable COPD group than in the healthy group, and in the stable COPD group, lymphocyte counts were also significantly lower in the death group than in the survival group [16]. Sorensen enrolled 386 patients with moderate to very severe COPD in stable phase without systemic treatment with glucocorticoids and found that lymphopenia was an independent predictor of all-course mortality [17]. Nevertheless, the study populations in these articles were COPD patients in a stable phase, which is different from the population we studied. To our knowledge, our study is the first to focus on the prognostic outcome of lymphocytopenia in patients with severe AECOPD.

Lymphocytes are one of the inflammatory markers, and different subpopulations of lymphocytes are involved in different stages of COPD development [18]. COPD leads to the activation of the adaptive immune system of the body, causing lymphocytes to infiltrate the small airways of the lungs [9], which may cause fewer circulating lymphocytes. A Spanish survey showed that early COPD patients had significantly lower lymphocyte counts than the non-COPD population [19]. AECOPD is associated with both systemic and pulmonary inflammation and is accompanied by elevated inflammatory mediators and multiple inflammatory cell changes [20]. One study found that compared to the control group, lymphocytes were lower in patients with stable COPD and lowest in patients with AECOPD (237.9 ± 76.2 vs. 193.6 ± 105.6 vs. 143.2 ± 68.5, *p* < 0.001) [21]. On the other hand, low lymphocyte count is often associated with old age and malnutrition, which is consistent with the clinical features of patients with COPD [22]. As our study shows here, patients with low lymphocytes are on average older and have lower albumin. As a sign of impaired immunity, decreased lymphocytes mean that patients are more susceptible to infections, a major cause of death in patients with severe AECOPD. In addition, a study analyzing patients admitted to the ICU found that patients in the non-survivor group had a significantly lower lymphocyte count compared to surviving patients [23]. This may be related to the fact that critically ill patients in ICU tend to have more severe infections. AECOPD is often accompanied by bacterial or viral infections, and the infections may lead to apoptosis of lymphocytes. All in all, the mechanisms underlying low lymphocytes in patients with AECOPD have not been fully elucidated and still need to be explored in more studies.

People have learned that low lymphocyte counts are associated with poor prognosis in numerous acute and chronic diseases such as sepsis [24], cancer [25] and cardiovascular disease [26]. This phenomenon is often seen in serious MERS (Middle East respiratory syndrome) infections as well [27]. In the global epidemic of COVID-19 in recent years, researchers have also found that lower lymphocyte count is prevalent in patients. In hospitalized COVID-19 patients, peripheral blood lymphocyte count was reduced, as were the counts of various lymphocyte subgroups [28]. The predictive value of lymphocytes in humans needs to be explored in more studies.

This study population consisted of patients with severe AECOPD, and to our knowledge, this is the first to focus on the predictive role of lymphocytes on poor prognosis in patients with severe AECOPD. We also found that patients with low lymphocyte had significantly longer hospital stay and time on ventilator compared to patients with normal lymphocyte count. Few studies have focused on the relationship between lymphocyte count and these two outcomes. As previously mentioned, among patients with severe AECOPD, a very high percentage of patients with low lymphocytes have respiratory failure (84.6%), which means that these patients are sicker and leads to longer hospital stays and use of ventilators.

Our study also had some limitations. First, our study was a single-center study with a small number of cases, lacking follow-up data on patients. Secondly, many patients failed to complete pulmonary function tests during this hospitalization due to the severity of the disease, and there were no CAT scores, mMRC scores or other indicators to assess the severity of patients, which prevented a more comprehensive prognosis of patients. Finally, interactions between lymphocyte subpopulations may have an impact on lymphocyte counts.

## Conclusion

In patients with severe AECOPD, lymphocytes < 0.8 × 10^9^/L was associated with higher in-hospital mortality.

## Supplementary Information

**Additional file 1: Table S1.** Clinical features and laboratory results between non-survivors and survivors. **Table S2.** Logistic regression analysis of the in-hospital mortality of severe AECOPD.

## Data Availability

The datasets during and/or analyzed during the current study available from the corresponding author on reasonable request.

## Abbreviations

COPD: Chronic obstructive pulmonary disease
AECOPD: Acute exacerbation of chronic obstructive pulmonary disease
OR: Odds ratio
CI: Confidence interval
BMI: Body mass index
IMV: Invasive mechanical ventilation
CHD: Coronary heart disease
CHF: Chronic heart failure
CKD: Chronic kidney diseases
GER: Gastroesophageal reflux
SAHS: Sleep apnea syndrome
NLR: Neutrophil/lymphocyte ratio
PLR: Platelet/lymphocyte ratio
CRP: C reactive protein
NT-proBNP: N-terminal pro-brain natriuretic peptide
PaO_2_: Arterial partial pressure of oxygen
PaCO_2_: Arterial partial pressure of carbon dioxide
MERS: Middle East respiratory syndrome
COVID-19: Coronavirus disease 2019
